# Paralogous *ALT1* and *ALT2* Retention and Diversification Have Generated Catalytically Active and Inactive Aminotransferases in *Saccharomyces cerevisiae*

**DOI:** 10.1371/journal.pone.0045702

**Published:** 2012-09-25

**Authors:** Georgina Peñalosa-Ruiz, Cristina Aranda, Laura Ongay-Larios, Maritrini Colon, Hector Quezada, Alicia Gonzalez

**Affiliations:** 1 Departamento de Bioquímica y Biología Estructural, División de Ciencia Básica, Instituto de Fisiología Celular, Universidad Nacional Autónoma de México, México City, México; 2 Unidad de Biología Molecular, Instituto de Fisiología Celular, Universidad Nacional Autónoma de México, México City, México; 3 Departamento de Bioquímica, Instituto Nacional de Cardiología, México City, México; SUNY at Buffalo, United States of America

## Abstract

**Background:**

Gene duplication and the subsequent divergence of paralogous pairs play a central role in the evolution of novel gene functions. *S. cerevisiae* possesses two paralogous genes (*ALT1/ALT2*) which presumably encode alanine aminotransferases. It has been previously shown that Alt1 encodes an alanine aminotransferase, involved in alanine metabolism; however the physiological role of Alt2 is not known. Here we investigate whether *ALT2* encodes an active alanine aminotransferase.

**Principal Findings:**

Our results show that although *ALT1* and *ALT2* encode 65% identical proteins, only Alt1 displays alanine aminotransferase activity; in contrast *ALT2* encodes a catalytically inert protein. *ALT1* and *ALT2* expression is modulated by Nrg1 and by the intracellular alanine pool. *ALT1* is alanine-induced showing a regulatory profile of a gene encoding an enzyme involved in amino acid catabolism, in agreement with the fact that Alt1 is the sole pathway for alanine catabolism present in *S. cerevisiae*. Conversely, *ALT2* expression is alanine-repressed, indicating a role in alanine biosynthesis, although the encoded-protein has no alanine aminotransferase enzymatic activity. In the ancestral-like yeast *L. kluyveri*, the alanine aminotransferase activity was higher in the presence of alanine than in the presence of ammonium, suggesting that as for *ALT1*, *LkALT1* expression could be alanine-induced. *ALT2* retention poses the questions of whether the encoded protein plays a particular function, and if this function was present in the ancestral gene. It could be hypotesized that *ALT2* diverged after duplication, through neo-functionalization or that *ALT2* function was present in the ancestral gene, with a yet undiscovered function.

**Conclusions:**

*ALT1* and *ALT2* divergence has resulted in delegation of alanine aminotransferase activity to Alt1. These genes display opposed regulatory profiles: *ALT1* is alanine-induced, while *ALT2* is alanine repressed. Both genes are negatively regulated by the Nrg1 repressor. Presented results indicate that alanine could act as *ALT2* Nrg1-co-repressor.

## Introduction

**Figure 1:**
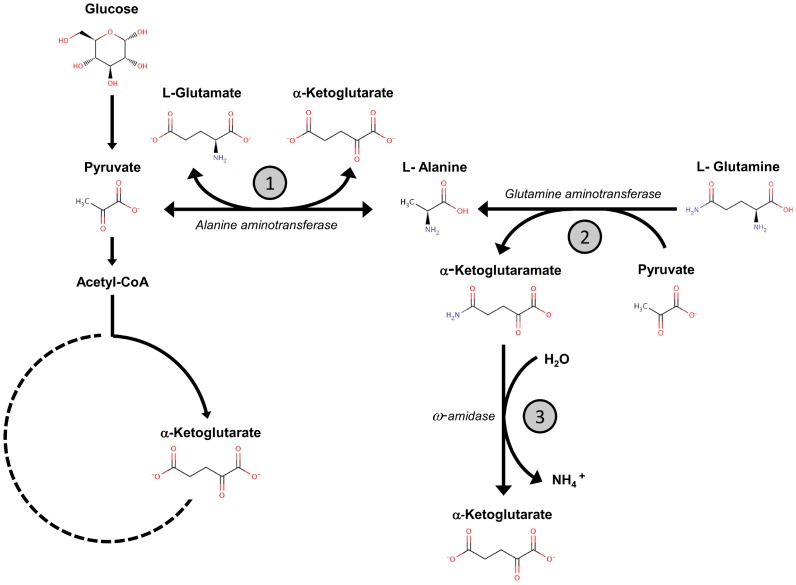
Model depicting pathways involved in alanine metabolism in Saccharomyces cerevisiae. Alanine is biosynthesized through the action of the *ALT1*-encoded aminotransferase (1). It is proposed that alanine is also synthesized through the irreversible action of glutamine aminotransferase (2), which belongs to the ω-amidase pathway, this enzyme catabolizes glutamine to α-ketoglutaramate leading to the formation of NH_4_ and α-ketoglutarate (3). Dashed lines indicate that TCA cycle enzymes are inactive when grown on glucose as the sole carbon source, except those leading to the replenishment of α-ketoglutarate.

Alanine aminotransferases (ALTS) are pyridoxal phosphate-dependent enzymes, which catalyze the reversible transamination between alanine and α-ketoglutarate to form pyruvate and glutamate (Figure 1). The presence of at least two alanine aminotransferase isozymes is widely distributed in animals, plants, yeasts and bacteria. In these systems, ALT isozymes have been considered to constitute the main pathway leading to alanine biosynthesis and catabolism [1]–[6]. Alanine catabolic and anabolic pathways are central in nitrogen and carbon metabolic networks. During growth under glucose-restricted conditions, alanine catabolism constitutes a key pathway for gluconeogenesis, since pyruvate can be readily converted to oxaloacetate through the action of pyruvate carboxylase, leading to glucose production [7]. In addition, it has been shown that in *Arabidopsis*, pyruvate anaerobic fermentation constitutes a metabolic mechanism that allows adaptation to low oxygen conditions. Under hypoxia/anoxia, pyruvate fermentation determines the synthesis of lactate, ethanol and acetaldehyde, thus favoring the regeneration of NAD^+^ from NADH. Energy shortage provoked by lower ATP yield due to the inactivation of oxidative phosphorilation is relieved by increasing NAD^+^ production and glycolytic rate [8]–[10]. Among other mechanisms that confer tolerance to hypoxia or anoxia, *Medicago truncatula* seedlings limit the accumulation of lactate to preserve the pH balance. In this regulatory circuit, alanine biosynthesis plays a crucial role, since it restricts the utilization of pyruvate to form lactate or ethanol, preventing acidification by lactate and shortage in carbon availability [11]–[12].

Differences in structure, kinetic parameters, tissue and subcellular distribution and gene regulation of ALT isozymes suggest that the two isoforms could have opposed tendencies for either alanine or pyruvate synthesis in mammals [4]. In fact, it has been found that mitochondrial ALT isozyme (m-ALT) expressed in gluconeogenic tissues, like liver and kidney, is involved in alanine catabolism. In glucogenic tissues like skeletal and cardiac muscle, ALT is cytosolic (c-ALT) and participates in alanine biosynthesis [13]. During starvation c-ALT synthesizes alanine as a consequence of amino acid degradation in glucogenic tissues. It is also known that alanine travels through the bloodstream and in the liver it is transaminated by m-ALT to yield pyruvate, which is converted to glucose [7].

No orthologous counterparts of the bacterial alanine biosynthetic enzymes, alanine dehydrogenase (AlaD) [14]–[15] or aspartate-4-decarboxylase (AspD) [16] have been found in fungal systems; it was thus considered that in fungi the main biosynthetic pathway leading to alanine production would be constituted by ALT isozymes. Since the action of ALTS is reversible and no other alanine catabolic enzyme has been found in yeast, it was assumed that ALTS would constitute the sole pathway for alanine catabolism. Purification and biochemical characterization of ALT from *Candida maltosa* showed that this enzyme has high affinity towards alanine, suggesting that it could play a catabolic function [5]; its role in alanine biosynthesis has not been determined.

In *S. cerevisiae*, two paralogous genes *ALT1* and *ALT2*, have been found and it was proposed that these genes encode alanine aminotransferases determining alanine biosynthesis and catabolism. This paralogous pair forms part of a duplicated chromosomal block generated from the Whole Genome Duplication (WGD) event [17], [18] (http://www.gen.tcd.ie/~kwolfe/yeast/nova/index.html). An additional inspection using the Yeast Gene Order Browser (http://wolfe.gen.tcd.ie/ygob/) [19] also suggests that *ALT1/ALT2* are in a duplicated block. This evidence points to the origin of the *ALT1-ALT2* duplicated gene pair as part of the WGD duplication event rather than to an isolated gene duplication phenomenon.

Previous results from our laboratory showed that simultaneous *ALT1* and *ALT2* impairment did not result in alanine auxotrophy in media containing glucose as sole carbon source;, indicating the existence of an alternative pathway for alanine biosynthesis [20]. Although these results do not discard the possibility that Alt1 and Alt2 could play a role in alanine biosynthesis, the existence of at least one additional pathway must be surmised. Accordingly it was proposed that the pathway could be afforded by glutamine-pyruvate aminotransferase. This enzyme forms part of the ω-amidase pathway [21]–[25], which has been considered to play a biosynthetic role; it cannot provide a catabolic pathway, since the reaction catalyzed by glutamine aminotransferase is irreversible (Figure 1) [24], [25]. In regard to the role of Alt1 and Alt2 in alanine catabolism, it was found that *alt1*Δ mutants do not grow on alanine as sole nitrogen source. Because *alt2*Δ mutants have no evident phenotype, it was concluded that Alt1 displayed both biosynthetic and catabolic capacities. Accordingly, *ALT1* expression was found to be alanine-induced, again suggesting a catabolic character. It is noted that neither *ALT2* expression, nor enzymatic activity was detected under the conditions tested [20].

Analysis of metabolic flux in fourteen hemiascomycetous yeasts revealed that most species that diverged after the genome duplication contain both ALT isoforms. As mentioned earlier, both biosynthetic and catabolic roles have been assigned to Alt1, whereas the physiological role of Alt2 has not been ascertained. Earlier divergent species contained a unique alanine aminotransferase, showing high identity with *ALT1* of *S. cerevisiae* [26].

This paper addresses the questions of whether *ALT2* encodes an active alanine aminotransferase, and whether Alt1 and Alt2 have diversified their in vivo physiological role. Our results indicate that: i) the *ALT2* encoded enzyme does not bear pyruvate-alanine aminotransferase activity. Its retention suggests it could have an alternative function, ii) that *ALT1* and *ALT2* gene expression profiles have diversified, that iii) as expected for earlier yeast divergent species, LkAlt1 has an enzymatic activity profile similar to that observed for Alt1, and iv), that *ALT1* and *ALT2* gene expression is regulated through differential Nrg1-dependent repression.

## Results

### Alt1 Constitutes the Main Pathway for Alanine Biosynthesis

**Figure 2:**
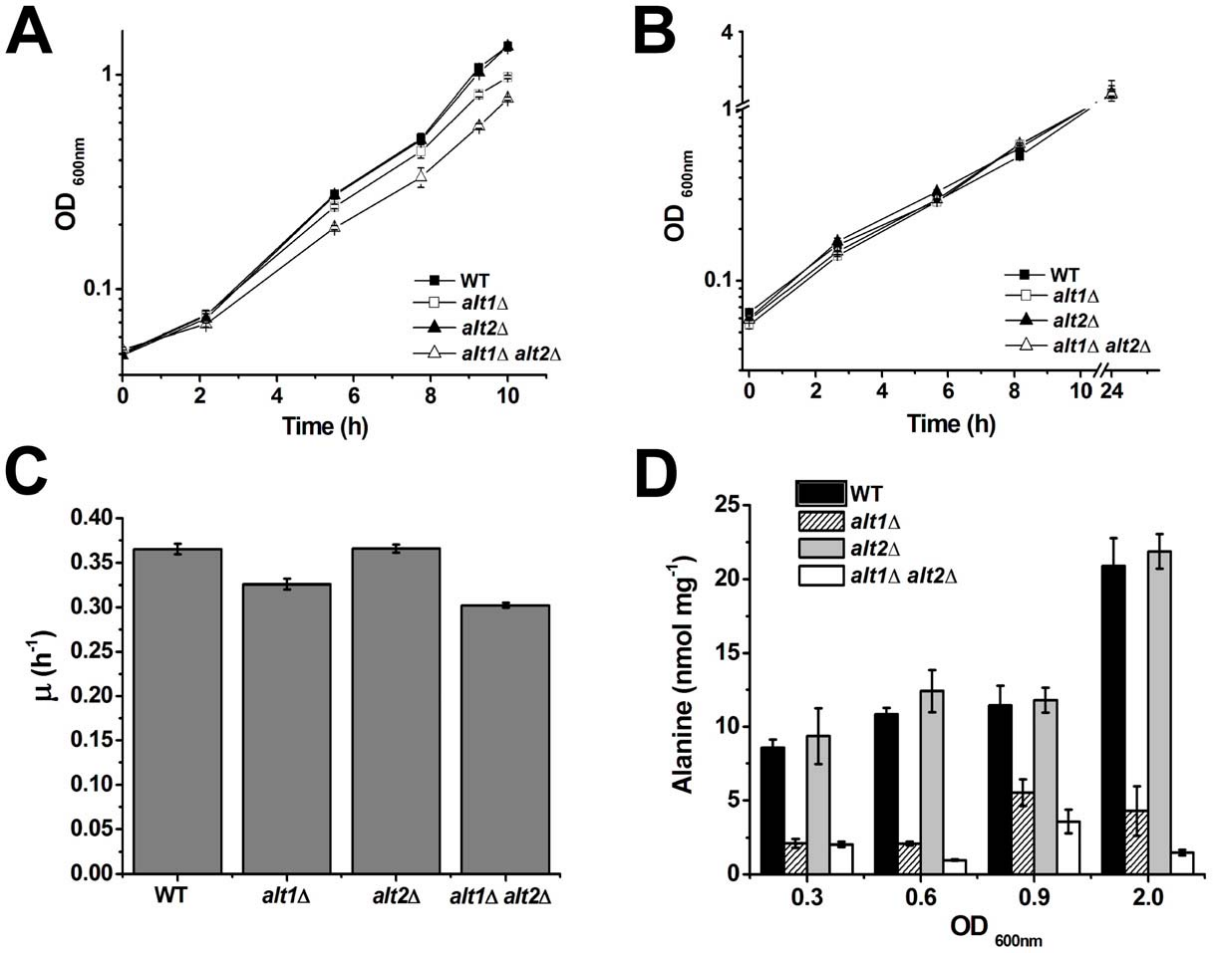
Single *alt1*Δ and double *alt1*Δ *alt2*Δ mutants display alanine partial auxotrophy. Wild type, *alt1*Δ, *alt2*Δ and *alt1*Δ *alt2*Δ were grown on ammonium-glucose (A) or alanine-ammonium-glucose (B). Graphed values represent means of three independent experiments ± SD. Specific growth rate was determined during exponential phase in glucose-ammonium cultures (C). Values are presented as means ± SD from three independent experiments. Intracellular concentration of alanine in extracts obtained from glucose-ammonium-grown cells (D). Yeast cells were grown and harvested when the cultures reached the stated optical density. Cell–free extracts were prepared and alanine pools were determined as described in Material and methods.

Previous work from our laboratory indicated that when glucose is present as sole carbon source, an *ALT1-ALT2* independent pathway plays an important role in alanine biosynthesis, since double *alt1*Δ *alt2*Δ mutants show wild type growth rate [20]. To analyze Alt1 and/or Alt2 contribution to alanine biosynthesis, single and double mutants were grown on ammonium-glucose with or without alanine (Figure 2A and 2B). It was found that in both conditions, the wild type strain and *alt2*Δ mutant showed equivalent growth rates, indicating alanine prototrophy. However, the *alt1*Δ mutant showed a lower growth rate on glucose ammonium than that displayed by the wild type strain (0.31 vs 0.36 h^−1^; Figure 2C). The *alt1*Δ *alt2*Δ double mutant showed lower growth rate than the wild type strain (0.28 vs 0.36 h^−1^) and than the *alt1*Δ single mutant (0.28 vs 0.31 h^−1^; Figure 2C). Single and double mutants grew as well as the wild type strain in the presence of alanine, indicating that *alt1*Δ and double *alt1*Δ *alt2*Δ mutant require alanine to achieve wild type phenotype. Growth rates between the wild type strain and the single *alt1*Δ mutant, and between the single *alt1*Δ and double mutant strains were significantly different (*P* value <0.01). These results suggest that both, Alt1 and Alt2 participate in alanine production.

Alanine pools were determined in the wild type, single mutant and double mutant strains. During early exponential and late exponential growth phases (OD_600_ 0.3, 0.6 and 0.9) Alt1 contributed 75–60% to the alanine pool, whereas the Alt1-Alt2 independent pathway(s) provided only 25–40%. Alt2 contribution seems negligible because no difference in the alanine pool was observed between the WT and the *alt2*Δ strain. The absence of *ALT1* (single *alt1*Δ and double *alt1*Δ *alt2*Δ) evidenced the contribution of the Alt1-Alt2 independent pathway(s). During stationary phase (OD_600_ 2.0) Alt1 afforded 82%, while the Alt1-Alt2-independent pathway(s) contributed 6%. Only under this condition, Alt2 contribution (12%) became significant, since *alt1*Δ mutant showed higher alanine pool than the double *alt1*Δ *alt2*Δ mutant; the positive role of Alt2 was only observed in an *alt1*Δ genetic background (Figure 2D). The deletion of an enzyme-encoding gene may have other effects on metabolic pathways besides the reduction in the corresponding enzymatic activity [27]. Nevertheless, in the *alt1*Δ mutant, the alanine pool decreased 80% as compared with the wild type strain, indicating that Alt1 plays an important role in the building up of the alanine reservoir. Moreover, the fact that single *alt2*Δ mutants showed a growth rate and an alanine pool equivalent to that found in the wild type strain, indicates null Alt2 contribution in an otherwise wild type strain.

### *ALT1* and *ALT2* are Differentially Expressed Under Biosynthetic or Catabolic Conditions

**Figure 3:**
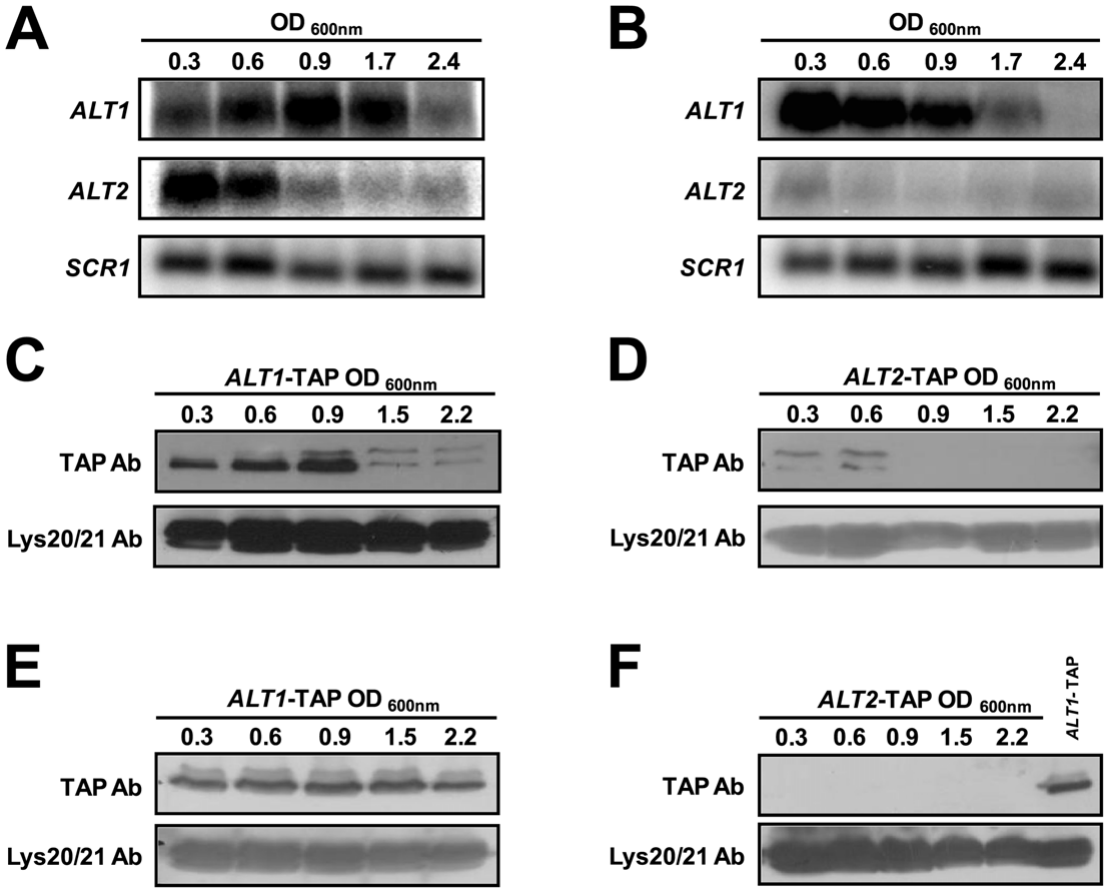
*ALT1* and *ALT2* display differential gene expression pattern, which is consistent with Alt1-Alt2 intracellular concentration. Northern blot of total RNA prepared from wild type strain grown on either glucose-ammonium (A) or glucose-alanine (B). Samples were collected from various OD_600_ as stated. Representative results from three experiments are shown. Cell-free extracts from *ALT1*-TAP (C and E) or *ALT2*-TAP (D and F) tagged strains grown on glucose-ammonium (C and D) or glucose-alanine (E and F) were prepared and subjected to immunoblot analysis using anti-TAP polyclonal antibody. As a loading control, each nitrocellulose membrane was also subjected to immunoblot analysis using anti-Lys20/Lys21 antibody. All lanes were loaded with 100 µg of protein. An *ALT1*-TAP sample was included as a loading control in panel F. Results are representative of three independent experiments.

Northern blot analyses were carried out in samples obtained throughout different growth phases attained on glucose-ammonium (Figure 3A). *ALT1* expression reached its highest peak during late exponential phase (OD_600_ 0.9) which was followed by a decrease in expression during stationary phase. The increase in ALT1 expression is consistent with the higher contribution of Alt1 to the alanine pool during stationary phase OD_600_ 2.0 (Figure 2D). Conversely, low expression of *ALT1* observed at OD_600_ 0.3 and 0.6 reflects the low Alt1 contribution to the alanine pool at OD_600_ 0.9 (Figure 2D). *ALT1* and *ALT2* expression patterns on glucose-ammonium (Figure 3A) suggest that the endogenous alanine pool could simultaneously determine *ALT1* induction and *ALT2* repression. *ALT2* expression was only observed during early exponential phase, indicating that Alt2 could contribute to alanine biosynthesis during early exponential growth; however, *alt2*Δ mutant shows alanine pools similar to those of the wild type strain.

**Figure 4:**
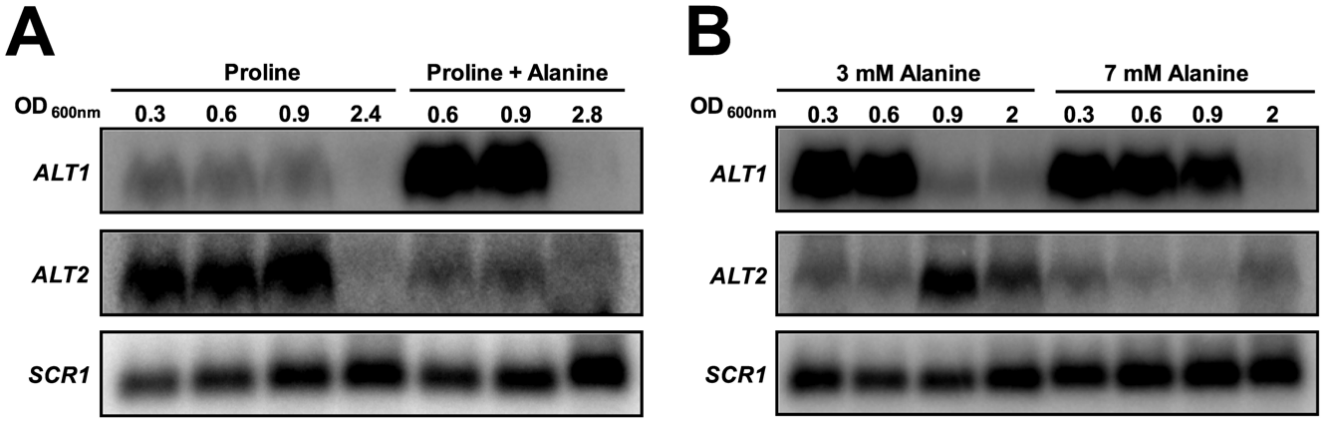
Alanine determines *ALT1* expression induction and *ALT2* expression repression. Northern blot of total RNA obtained from wild type strains grown on 7 mM proline with or without alanine (A) or on 3 mM or 7 mM alanine (B), as nitrogen sources.

*ALT1* and *ALT2* expression was monitored on samples obtained from glucose-alanine grown cultures (Figure 3B). *ALT1* mRNA induction was observed throughout the exponential phase. In the pre-stationary and stationary phases relatively low *ALT1* levels were detected. In contrast, *ALT2* expression was repressed throughout the different growth phases, suggesting Alt2 role in alanine biosynthesis. These results confirm previous observations indicating that *ALT1* expression pattern is characteristic of a gene encoding a catabolic enzyme [20]. Accordingly, an *alt1*Δ *ALT2* mutant is unable to grow on alanine as sole nitrogen source [20], showing that Alt2 cannot complement Alt1catabolic role. To further analyze this issue, cultures were grown on 7 mM proline as sole nitrogen source to mid-exponential phase; the culture was then divided: half of it was allowed to continue growth on proline, 7 mM alanine was added to the rest of the culture. Aliquots for RNA extraction were taken at different OD_600 nm_ and Northern analysis was performed. Figure 4A shows that after alanine addition, *ALT1* expression increased, while *ALT2* was repressed, indicating that alanine influences *ALT1* and *ALT2* expression profile. Furthermore, when Northern analysis was performed on total RNA samples obtained from cultures grown on two different alanine concentrations, *ALT1*-dependent induction was transiently observed when 3 mM alanine was added (OD_600 nm_ 0.3–0.6). However, when 7 mM alanine was present in the medium, *ALT1* induced expression was observed for a longer period (OD_600 nm_ 0.3–0.9; Figure 4B), suggesting that differential alanine exhaustion from the media affected *ALT1* induction. As expected, *ALT2* expression showed a reciprocal behavior (Figure 4B).

### Alt1 and Alt2 Protein Concentration is Consistent with *ALT1* and *ALT2* Expression Profile

Immunoblots were carried out with *ALT1*-TAP and *ALT2*-TAP derivatives as described in methods. Both, Alt1 and Alt2 protein concentrations were consistent with mRNA expression profiles. In glucose-ammonium cultures, increased *ALT1* transcription resulted in higher Alt1 concentration, whereas Alt2 was only detected during the early exponential growth phase (Figure 3C and 3D). As expected, in extracts obtained from glucose-alanine cultures, Alt2 was not detected, whereas Alt1 was present throughout the various growth phases (Figure 3E and 3F). Figure S1A and S1B shows that *ALT1*-TAP and *ALT2*-TAP displayed a similar expression profile to that observed in the CLA1 strain collection, shown in Figure 3A and B.

### Nrg1 Determines *ALT1* and *ALT2* Expression Repression

The overall results indicate *ALT1* and *ALT2* display differential expression patterns on glucose with either ammonium or alanine as nitrogen sources: alanine induces *ALT1* expression and represses that of *ALT2*. Additionally it was found that *ALT1* expression is repressed during pre-stationary and stationary growth phases.

**Figure 5:**
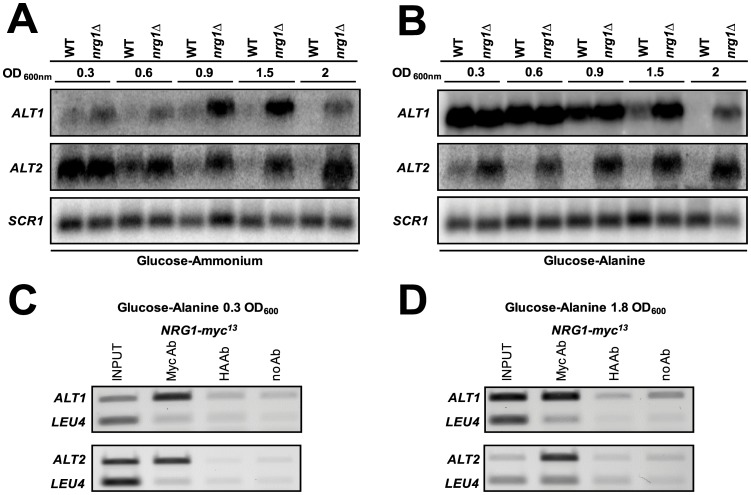
Nrg1 determines *ALT1* and *ALT2* repression. Northern blot of total RNA obtained from wild type and *nrg1*Δ strains grown on either glucose-ammonium (A) or glucose-alanine (B). Samples were collected from various OD_600_ as stated. Representative results from three experiments are shown. ChIP assays were performed using anti-Myc antibody on wild type strains containing myc^13^ epitope-tagged *NRG1*. As a control, equivalent samples were treated with anti-HA antibody. Strains were grown on 2% (w/v) glucose +7 mM alanine to either 0.3 or 1.8 OD_600_, cells were centrifuged, collected and used to prepare samples for ChIP (C, D). PCR was performed with the deoxyoligonucleotides described in Table 3. As a negative control, PCR was also performed for *LEU4* coding region. Results are representative of three independent experiments.

Multiple sequence alignment of *ALT1* and *ALT2* promoters present in four *Saccharomyces* ¨sensu strictö,identified a consensus binding sequence for the *NRG1*-encoded repressor in each promoter (Figures S2, S3 and S4). To examine if Nrg1 was involved in repression, *nrg1*Δ mutants were constructed and Northern blot analysis was carried out. It was found that both, *ALT1* and *ALT2* expression was Nrg1-repressed (Figure 5A and 5B). To determine Nrg1 occupancy of the *ALT1* and *ALT2* promoters, chromatin immunoprecipitation (ChIP) was performed in extracts obtained from glucose-alanine grown cultures. Nrg1 was bound to the *ALT1* promoter at OD_600nm_ 0.3, when expression was induced, and at OD _600 nm_ 1.8 when repression was observed; this suggests that Nrg1 is permanently bound to the *ALT1* promoter. Nrg1 was bound to the *ALT2* promoter in both exponential and stationary growth phases (Figure 5C and 5D).

Phenotypic analysis of null *nrg1*Δ mutants showed that on glucose-ammonium, these strains displayed a lower growth rate, compared with the *NRG1* wild type strain (0.11 *vs* 0.20 h^−1^). The effect on growth rate observed in the *nrg1*Δ mutants cannot be solely attributed to changes in *ALT1* and *ALT2* expression, since Nrg1 is a global transcriptional modulator which mediates glucose repression and negatively regulates a variety of processes [28]. On glucose-alanine, the wild type strain showed a growth rate of 0.15 h^−1^, while the *nrg1*Δ mutant strain had a growth rate of 0.05 h^−1^. The single and double *alt1*Δ and *alt1*Δ *nrg1*Δ mutants were unable to grow on alanine as sole nitrogen source, showing that even under derepressive conditions, Alt2 was unable to complement the lack of Alt1 (data not shown).

### *ALT2*-encoded Protein does not Display Alanine Aminotransferase Activity

**Figure 6:**
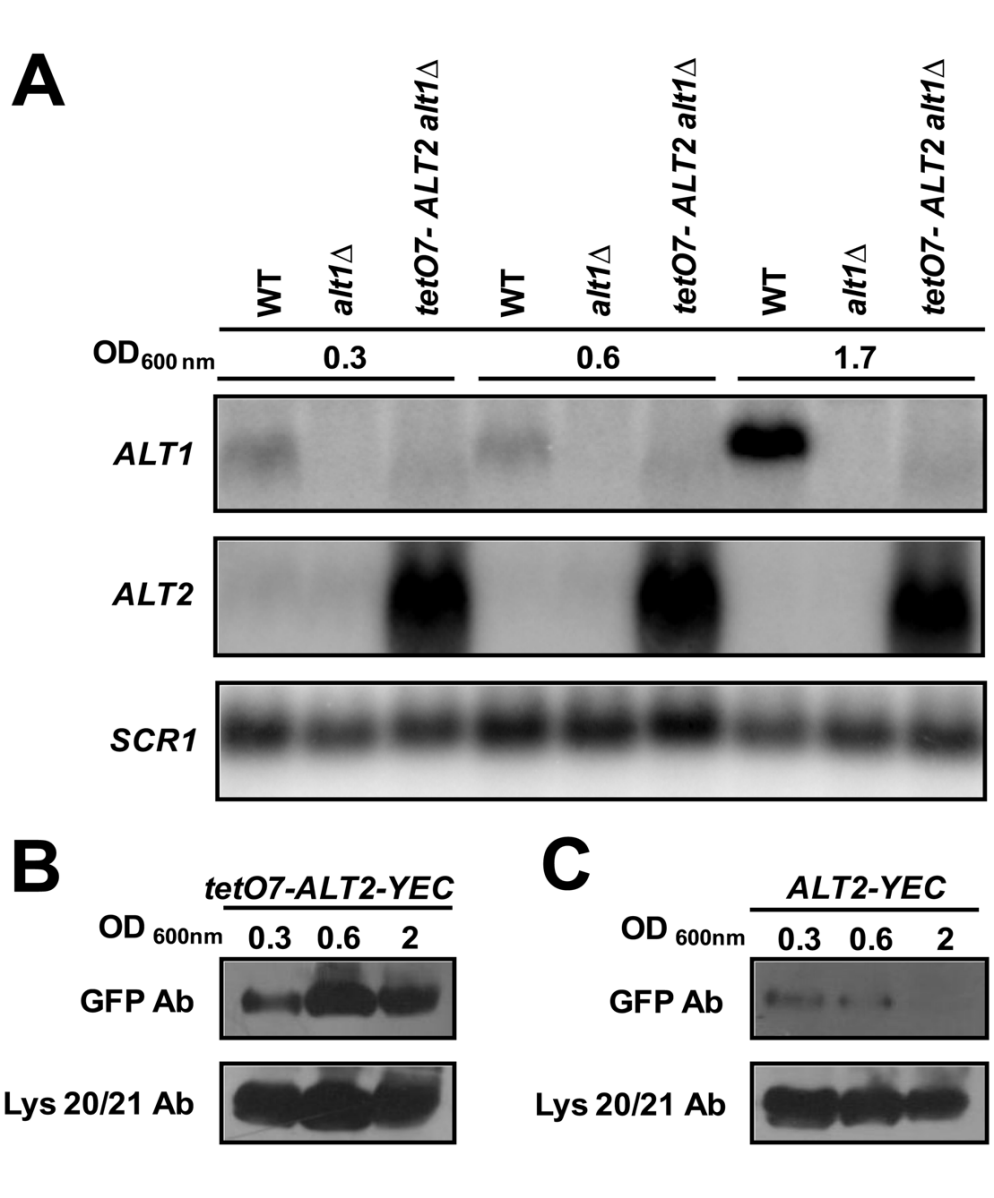
*tetO7* promoter increases *ALT2* expression, which is consistent with Alt2 intracellular concentration. Northern blot of total RNA prepared from wild type strain, *alt1*Δ and *alt1*Δ *tetO7-ALT2* grown glucose-ammonium (A). Samples were collected from various OD_600_ as stated. Representative results from three experiments are shown. Cell-free extracts from *tetO7-ALT2-yEcitrine (tetO7-ALT2-YEC)* (B), and *ALT2-yEcitrine (ALT2-YEC)* (C) tagged strains grown on glucose-ammonium were obtained and subjected to immunoblot analysis using anti-GFP monoclonal antibody. As a loading control, nitrocellulose membranes were also immunoblotted with anti-Lys20/Lys21 antibody. All lanes were loaded with 100 μg of protein.

To increase *ALT2* expression, *ALT2* promoter was substituted by the bacterial *TET* promoter in the R1158-3 *alt1*Δ *ALT2* background, generating *alt1*Δ *tet07*-*ALT2* strain. Figure 6A shows that when RNA was prepared from cultures grown on glucose-ammonium, transcription from the *TET* promoter increased *ALT2* expression. C-tagged strains *ALT1 tetO7*-*ALT2-yECitrine* and *ALT1*-*ALT2*-*yECitrine* were constructed; Alt2 was immunodetected in both strains. As expected, Alt2 concentration was higher in the *tetO7*-*ALT2*-*yECitrine* mutant than in the *ALT1*-*ALT2*-*yECitrine* control strain, thus indicating that a relatively high *ALT2*-expression was accompanied by higher protein synthesis (Figure 6B and 6C).

Alt2 enzymatic activity was assayed in *alt1*Δ *tet07*-*ALT2*, and *alt1*Δ *nrg1*Δ extracts of cultures grown on ammonium-glucose to an OD_600 nm_ of 1.0. No activity was detected, suggesting that Alt2 was completely devoid of alanine aminotransferase enzymatic capacity. Furthermore *alt1*Δ *tet07*-*ALT2* did not grow on glucose alanine, showing that *ALT2*-encoded protein was unable to complement Alt1 lack. Background activity values from *alt1*Δ mutants (Table 1), cannot be attributed to alanine aminotransferase, since the observed activity is practically identical, regardless of whether it was assayed in a strain displaying wild type *ALT2* levels (*alt1*Δ *ALT2* 0.019 µmol/min^−1 ^mg of protein^−1^) or an *ALT2* overexpressing mutant (*alt1*Δ *tet07-ALT2* 0.022 µmol/min^−1^ mg of protein^−1^). During stationary phase (OD_600 nm_ 2.0), and in the absence of Alt1, Alt2 apparently exerted a small positive effect over alanine intracellular pool (Figure 2D).The fact that an *alt1*Δ *alt2*Δ mutant showed a lower alanine pool than that of the *alt1*Δ strain indicates that Alt2 could play a role as a positive modulator of the enzyme (s) contributing the alternative pathway(s) of alanine biosynthesis. However a direct role of Alt2 in alanine biosynthesis can be excluded.

**Table 1:** Alanine aminotransferase specific activity in extracts prepared from glucose-NH_4_ grown cultures.

Strains	Specific activity
CLA1-2 (*ALT1 ALT2*)	0.104±0.030
CLA1-2-1 (*alt1*Δ *ALT2*)	0.011±0.003
CLA1-2-2 (*ALT1 alt2*Δ)	0.117±0.030
CLA1-2-D (*alt1*Δ*-alt2*Δ)	0.023±0.000
CLA 603 (*alt1*Δ*-ALT2 nrg1*Δ)	0.015±0.004
R1158 (*ALT1 ALT2)*	0.130±0.060
R1158-3 (*alt1*Δ *ALT2*)	0.019±0.008
R1158-4 (*ALT1 tetO7-ALT2*)	0.110±0.040
R1158-5 (*alt1*Δ *tetO- ALT2*)	0.022±0.003

Specific activity is expressed as µmol/min^−1^ mg of protein^−1^. Values are presented as mean from at least three measurements ± S. D.

**Figure 7:**
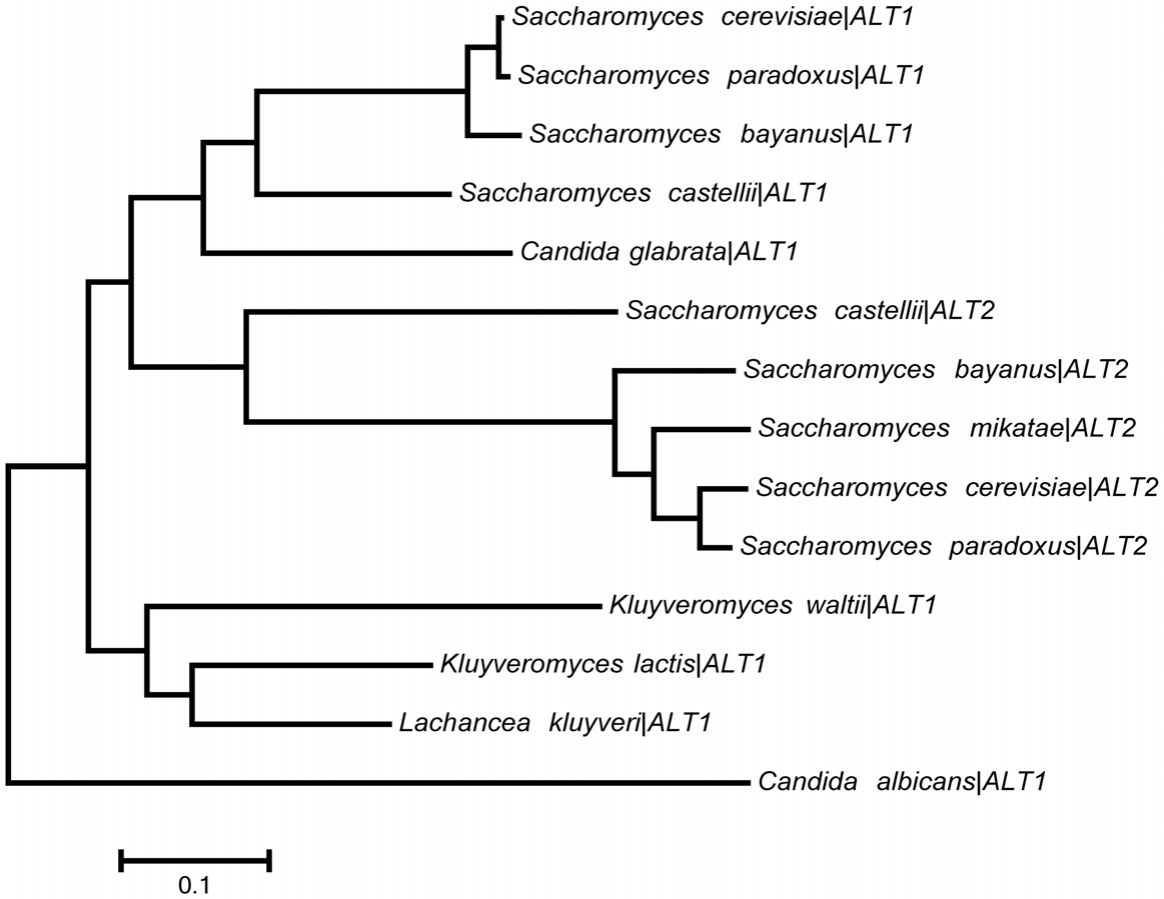
Figure. Evolutionary relationships of alanine aminotransferases present in post-WGD yeasts. The evolutionary history was inferred using the Maximum-Likelihood method [29]. The branch lengths are measured in the number of substitutions per site. *ALT1* and *ALT2* are clustered in different subtrees with their corresponding post-WGD yeasts orthologues. Since *ALT2* branch is longer than *ALT1* branch, a higher divergence rate for *ALT2* is suggested.

A phylogenetic study of *ALT1-ALT2* in pre- and post-WGD yeasts, showed that the ancestral like yeasts (*L. kluyveri, Kluyveromyces. lactis* and *Kluyveromyces waltii*) constitute a group which originated both *ALT1* and *ALT2*, supporting the WGD origin of these genes. Figure 7 shows that *ALT2* and *Saccharomyces* “sensu strico” orthologues form a subtree with longer branches than *ALT1* orthologues, which are clustered in a different subtree. This result suggests that Alt2 has diverged as consequence of a high substitution rate whereas Alt1 has undergone few modifications. To further analyze this possibility, alanine aminotransferase activity was determined in extracts obtained from cultures of the *L. kluyveri* wild type strain grown on glucose + ammonium or on glucose + ammonium + alanine. It was found that, as well as for the *S. cerevisiae ALT1*-encoded isozyme [20], *Lk*Alt1 alanine aminotransferase specific activity increased almost ten-fold in the presence of alanine (0.450±0.007 µmol/min^−1^ mg of protein^−1^), compared with the activity found on ammonium (0.049±0.009 µmol/min^−1^ mg of protein^−1^). These results indicate that the single orthologue present in *L. kluyveri* encodes an active alanine aminotransferase with an activity profile similar to that observed for Alt1. *ALT1* and *ALT2* have diverged, and since *ALT2* branch is longer it could be considered that the encoded-enzyme has evolved a new function (Figure 7) [29]–[31]. However a physiological analysis would be necessary to test this possibility.

To determine whether loss of Alt2 aminotransferase activity could be due to mutations in catalytic amino acids, a multiple alignment of the Alt proteins from *S. cerevisiae* and post WGD yeasts, was performed. It was found that the catalytic residues involved in cofactor and substrate binding have been conserved in all cases [32]. (Figure S5). These results suggest that lack of Alt2 activity could be due to mutations affecting oligomeric organization or other amino acids playing a critical role in enzyme structure and catalytic capacity.

## Discussion

This paper addresses the roles of Alt1 and Alt2 in alanine metabolism in *S. cerevisiae*. The results show i) that in glucose-grown cells, Alt1 is instrumental in alanine catabolism, and contributes from 60 to 80% of the alanine intracellular pool and ii) that Alt2 does not display alanine aminotransferase activity and does not complement Alt1 lack. These data indicate that the biosynthetic and catabolic capacity displayed by alanine aminotransferases has been delegated to Alt1, and that *ALT2*-encoded protein does not participate in alanine metabolism, thus explaining Alt2 incapacity to complement Alt1 lack. The existence of a strictly biosynthetic pathway would have left *ALT2* free to evolve new functions, while *ALT1* retention would be favored, since it constitutes the sole pathway for alanine catabolism. It has been proposed that alanine biosynthesis could be achieved through the action of the glutamine aminotransferase, an irreversible pathway, unable to participate in alanine catabolism [20], [24], [25] (Figure 1).

### Nrg1 Determines Negative *ALT1* and *ALT2* Transcriptional Regulation

*ALT2* expression is repressed in the presence of alanine, suggesting that this amino acid could act as Nrg1 co-repressor. After alanine exhaustion, Nrg1 determines *ALT1* repression, suggesting that for this gene, the co-repressor could be a product of alanine catabolism. This system could function as a genetic switch, ensuring that gene activity is turned off when the end product is available. The negative regulation of the genes involved in alanine metabolism could be determined by Nrg1-containing complexes, which could alternatively determine repression of either *ALT1* or *ALT2* depending on the intracellular content of either alanine or a product of its catabolism. In fact, arginine metabolism constitutes an example in which small substrate molecules serve as signals directly influencing transcription [33]. In the presence of arginine, the ArgII-ArgI-Mcm1 heteromeric complex binds to its cognate site in the pertinent promoters repressing expression of biosynthetic genes, and preventing binding of the CARGR repressor, thus allowing expression of CargA and CargB catabolic genes [34]. This system facilitates biosynthesis repression and catabolism induction in the presence of arginine.

Since Alt1 has retained both the biosynthetic and catabolic character of alanine aminotransferase, it could be suspected that Alt1 could catalyze futile cycles. However the fact that *ALT1* expression is activated in the presence of alanine and repressed after alanine consumption indicates that the combined action of induction-repression mechanisms could contribute to the avoidance of such cycles. For example, in the case of the Bat1 and Bat2 branched chain aminotransferases, functional divergence through differential gene expression, has resulted in the asymmetric distribution of the biosynthetic and catabolic character between Bat1 and Bat2 [35]. The independent regulation of each gene, selectively determines the presence of the pertinent isozymes under either biosynthetic or catabolic conditions [35].

### Why has *ALT2* been Retained?

Although Alt2 shows no alanine aminotransferase activity, its retention as well as its characteristic expression profile, suggest that Alt2 could have originally played a biosynthetic role. This could have been lost as consequence of mutation accumulation in the coding region; however it is noteworthy that the promoter has retained the capacity to respond to alanine concentration. Hence Alt2 could be a monitor of alanine biosynthesis by modulating other gene products. Alternatively, it could be considered that *ALT2* plays an additional physiological role not related to alanine metabolism, as it has been shown for the *S. cerevisiae* Lys20 paralogous enzyme. In addition to the role in lysine biosynthesis through homocitrate synthase activity (HCS) [36] Lys 20 is also involved in the process of DNA damage repair [37]. Whether Lys20 paralogous couple (Lys21) is also a bifunctional protein remains to be established. Nonetheless the case of Lys20-Lys21 illustrates that retention of bifunctional duplicated genes could involve diversification of the two functions carried out by the paralogous proteins. Alt2 forms synthetic lethals with various and unique partners [38], [39] which suggest that it could have alternative roles not related to that of alanine aminotransferase [38], [39]. It could also be considered that some of *ALT2* unique interactors (*UBP15*) could form part of a regulatory circuit influencing alanine metabolism or a more general adaptation response [40]. In this regard, the fact that *ALT2* expression is determined through an Nrg-dependent regulation may indicate that the encoded products could play a role in the adaptation to environmental stress [41].

### Concluding Remarks

Retained duplicate genes either provide an increased dosage of the same product; or they undergo a process of subfunctionalization or neofunctionalization, during which both copies of the gene loose a subset of their ancestral functions, and acquire new properties. In previous studies, we showed that subfunctionalization led to the retention of the genes encoding NADP^+^ glutamate dehydrogenase (*GDH1-GDH3*), and homocitrate synthase (*LYS20-LYS21*). In both cases, subfunctionalization resulted in the biochemical specialization of the encoded enzymes that led to balanced α-ketoacid utilization during growth on non-fermentable carbon sources, and the acquisition of facultative metabolism. Along the same line, subfunctionalization of the *BAT1* and *BAT2* genes encoding the branched chain aminotransferases (Bat1-Bat2) has primarily resulted in differential transcriptional regulation determining distribution of the biosynthetic and catabolic role of these enzymes [35], [36], [42]. The *ALT1-ALT2* case herein presented constitutes an example in which paralogous diversification has resulted in complete loss of the presumed ancestral function of one of the two copies, giving rise to a protein which, although being 65% identical to its active paralogous counterpart, is devoid of aminotransferase activity. A similar case has been described for the diversification of a bifunctional gene [43]. For this case, the dual character of the ancestral gene was distributed after duplication in two copies giving rise to *GAL3* whose product completely lost enzymatic properties and *GAL1* which conserved the galactokinase role [43]. The fact that *ALT2* encodes a protein with the expected molecular weight indicates that it is not a pseudogene. Thus, Alt1 and Alt2 physiological divergence poses the question of whether *ALT2* and *ALT1* functional diversification was the result of neofunctionalization or subfunctionalization. The fate of duplicated gene copies has been extensively discussed and several models have been proposed to account for duplicate conservation [44]. For the herein presented case of *ALT1/ALT2* subfunctionalization, it could be hypotesized that the ancestral gene could have encoded a bifunctional enzyme carrying the alanine aminotransferase function on one side and a yet undescribed second function on the other, and that subsequent diversification resulted in the distribution of each one of the two functions to Alt1 or Alt2. The fact that *Lk*Alt1 ancestral-like enzyme displays an activity profile similar to that of Alt1, suggests that the properties of the ancestral enzyme could have been delegated to Alt1. Determining whether *Lk*Alt1 is a bifunctional enzyme carrying out an additional function will allow to pose a neofunctionalization or a subfunctionalization model for *ALT1/ALT2* diversification.

## Materials and Methods

### Strains

Table 2 describes the characteristics of the strains used in the present work. Construction of strain CLA1-2 (*ura3 leu2::LEU2*) and its *alt1*Δ and *alt2*Δ derivatives has been previously described [20]. To obtain CLA1-2-1, CLA603 and R1158 *alt1*Δ derivatives, the pertinent strains were transformed with the 2612 bp PCR product containing the *kanMX4* cassette and *ALT1* upstream (756 bp) and downstream (263 bp) nucleotide sequences amplified from the genomic DNA of CLA1-2 strain, using deoxyoligonucleotides ALT1F and ALT1R (Table 3). The isogenic CLA1-602 *nrg1*Δ*::kanMX* was obtained from strain CLA1 by gene replacement [45]. A PCR-generated *kanMX4* module was prepared with plasmid pFA6a [45], using oligonucleotides NRG1F and NRG1R (Table 3).

**Table 2:** Strains used in this study.

Strain	Relevant genotype	Source
CLA1-2	*MATα ALT1 ALT2 ura3 leu3::LEU2*	[36]
CLA1-2-1	*MATα alt1*Δ*::kanMX4 ALT2 ura3 leu3::LEU2*	[20]
CLA1-2-2	*MATα ALT1 alt2*Δ*::kanMX4 ura3 leu3::LEU2*	[20]
CLA1-2-D	*MATα alt1*Δ*::kanMX4 alt2*Δ*::natMX ura3 leu3::LEU2*	[20]
CLA 602 *nrg1*Δ	*MATα ALT1 ALT2 ura3 leu3 nrg1*Δ*::nat MX4*	This study
CLA 603 *nrg1*Δ *alt1*Δ	*MATα ALT2 ura3 leu3 alt1*Δ *kan nrg1*Δ*:nat MX4*	This study
CLA1	*MATα ura3 leu2*	[36]
R1158 *ALT1 ALT2*	*MATa his3-1 leu2-0 met15-0 URA3::CMV-tTA ALT1 ALT2*	Open Biosystems
R1158-1 *ALT1 ALT2-yECitrine*	*MATa his3-1 leu2-0 met15-0 URA3::CMV-tTA ALT1 ALT2-yECitrine::HIS5*	This study
R1158-2 *ALT1 tetO7ALT2-yECitrine*	*MATa his3-1 leu2-0 met15-0 URA3::CMV-tTA ALT1 tetO7::kantMX4-ALT2-yECitrine::HIS5*	This study
R1158-3 *alt1*Δ *ALT2*	*MATa his3-1 leu2-0 met15-0 URA3::CMV-tTA alt1*Δ*::kanMX4 ALT2*	This study
R1158-4 *ALT1 tetO7-ALT2*	*MATa his3-1 leu2-0 met15-0 URA3::CMV-tTA alt1*Δ*::kanMX4 tetO7::natMX4-ALT2*	This study
R1158-5 *alt1*Δ*::kanMX4 tetO7-ALT2*	*MATα his3-1 leu2-0 met15-0 URA3::CMV-tTA alt1*Δ*::kanMX4 tetO7::natMX4-ALT2*	This study
*L. kluyveri* GRY1175	*MATα ura3*	[49]

Strain R1158 was obtained from Open Biosystems. R1158-1 derivative, *ALT2*-yECitrine was constructed by transforming strain R1158 with the *yECitrine::HIS5* cassette at *ALT2* C-terminus, generating strain R1158-1 (*ALT1 ALT2*-*yECitrine::HIS5*). A pair of deoxyoligonucleotides (ALT2F1 and ALT2R1) was designed based on the *ALT2* carboxy-terminal coding sequence, and that of the pKT175 *yECitrine::HIS5* multiple cloning site (Table 3). To construct strain R1158-2 *tetO7::kanMX-ALT2-yECitrine*, the chromosomal endogenous *ALT2* promoter of strain R1158-1 was replaced with a TET-titratable promoter *(tetO7*); the *tetO7* module was amplified from plasmid pCM325, which contains *KanR* as selectable marker [46]. A pair of deoxyoligonucleotides (ALT2-F2 and ALT2-R2) was designed based on the *ALT2* promoter sequence and that of the pCM325 multiple cloning site (Table 3). 2.2 Kbp PCR fragments were transformed into the R1158-1 yeast strain as previously reported [47]. The resulting strain *tetO7::kanMX4*-*ALT2* was transformed with the *yECitrine::HIS5* cassette, generating the *tet-O7*-*kanMX4*-*ALT2*-*yECitrine::HIS5* strain. The yECitrine cassette was amplified from plasmid pKT175-*HIS5*. The selection marker *URA3* from pKT175 was replaced by *HIS5* from the pKT101 plasmid. Correct module integration was PCR and sequencing confirmed.

**Table 3:** Oligonucleotides used for strain construction.

Primer	Oligonucleotide sequence	Application
ALT1F	5′-TCTTGCACGGTCGCATCTCTCG-3′	*ALT1* null mutant
ALT1R	5′-GATTAACGGGTGTTCGAACTACGC-3′	*ALT1* null mutant
NRG1F	5′-ATGTTTTACCCATATAACTATAGTAACCTCAATGTTTCTACTATGCCCGCACcgtacgctgcaggtcgac-3′	*NRG1* null mutant
NRG1R	5′-TTATTGTCCCTTTTTCAAATGTGTTCTATAGTGTTGCAAGCAATTATCATGatcgatgaattcgagctcg-3′	*NRG1* null mutant
ALT2F1	5′-TTCAAGACTGGAAAGAATTTCATCAAGATTTCTTCAGCAAGTATCGTAATggtgacggtgctggttta-3′	*ALT2-yECitrine*
ALT2R1	5′-ATGGATGCAAAACAAATAGAAAGCCATTATCATTAGTTTTTTTTTTTCATtcgatgaattcgagctcg-3′	*ALT2-yECitrine*
ALT2-F2	5′-CTATTGTTTCTACTAATGTGCTGCGGGCTGATGTCCTCCACACGTCTTCAcgtacgctgcaggtcgacgg-3′	*tetO7ALT2*
ALT2-R2	5′-AAATCCTTTGCGGTGAACACACCTTTCAAATCCTGTTGGTGTGTCATTGTcataggccactagtggatctg-3′	*tetO7ALT2*
NRG1F	5′-GATAATTGCTTGCAACACTATAGAACACATTTGAAAAAGGGACAAcggatccccgggttaattaa-3′	*NRG1-myc^13^*
NRG1R	5′-ATAGTAGTACTGCTAATGAGAAAAACACGGGTATACCGTCAATTAgaattcgagctcgtttaaac-3′	*NRG1-myc^13^*
ALT1Fw	5′ -AGACCCGTCCTACAGAGACATAGC-3′	*ALT1* Northern probe
ALT1Rv	5′ -GCGAGCTTCTTGAACTGCCTTGAA-3′	*ALT1* Northern probe
ALT2Fw	5′-GACACACCAACAGGATTTGAAAGG-3′	*ALT2* Northern probe
ALT2 Rv	5′-GCGTTCGCTTTCACAAAGAGCTT-3′	*ALT2* Norther probe
SCR1 Fw	5′-GGAGTTTTATCCAGGGTCAGCAAAGG3-′	*SCR1* Northern probe
SCR1 Rv	5′-TTTAATTTGGCGGTGCCATCAGGATTTA-3′	*SCR1* Northern probe

Lower-case lettering indicates sequence of the multiple cloning site of plasmid pFA6a *kanMX4* for null strain construction, pKT175 for yECitrine tagging, pCM325 for tet07 promoter fusion constructions and pFA6a-myc^13^-*kanMX6* for protein tagging.

Strain R1158-3 (*alt1*Δ *ALT2*) was constructed by transforming strain R1158 with the 2612 bp PCR product containing the *kanMX4* cassette and *ALT1* upstream (756 bp) and downstream (263 bp) nucleotide sequences amplified from the genomic DNA of the R1158 strain, using deoxyoligonucleotides ALT1F and ALT1R (Table 3).

Strain R1158-5 *alt1*Δ*::kanMX4 tetO7::natMX4-ALT2*, was derived from an R1158-4 *ALT1 tetO7::kanMX4-ALT2* obtained as described for R1158-2 construction. The *kanMX4* module from strain R1158-4 (*tetO7::kanMX4-ALT2*) was replaced by the *natMX21* cassette, which confers resistance to the antibiotic nourseothricin [48], generating strain *ALT1 tetO7::natMX21-ALT2.* The *natMX21* cassette used for transformation was obtained by digesting plasmid p4339 with EcoR1. To obtain *alt1*Δ derivative, strain *ALT1 tetO7::natMX21-ALT2* was transformed as previously described [20].

*ALT1*-TAP and *ALT2*-TAP were obtained from the TAP-tagged *Saccharomyces* strain collection.

The *Lachancea kluyveri* GRY1175 strain [49] kindly provided by Dr. Jure Piskur) was used to prepare extracts and determine alanine aminotransferase activity.

### Growth Conditions

Strains were routinely grown on MM containing salts, trace elements and vitamins following the formula of yeast nitrogen base (Difco). Filter sterilized glucose (2% w/v) was used as carbon source, and 40 mM ammonium sulphate or 0.1% (w/v) alanine were used as the nitrogen source. Amino acids needed to satisfy auxotrophic requirements were added at 0.01% (w/v). Cells were incubated at 30°C with shaking (250 r.p.m.).

*L. kluyveri* strains were grown in SC medium (containing 0.67% (w/v) yeast nitrogen base without amino acids and nitrogen source(Difco) and 2% (w/v) glucose) with 0.2% (w/v) NH2SO4 (SC+ammonium) or 0.1% (w/v) L-alanine (SC+alanine) as nitrogen sources. Uracile (20 mg/l) was added when required.

### Northern Blot Analysis

Northern analysis was carried out as previously described [50]. Total yeast RNA was prepared from 100 ml aliquots of cultures with the stated OD_600_ of MM with 40 mM (NH4)_2_SO_4_ or 0.1% alanine as nitrogen source and 2% glucose as carbon source. Three sets of deoxyoligonucleotides were used to PCR-amplify fragments that were used as probes for sequential hybridization of *ALT1* (1410 bp), *ALT2* (1335 bp) and SCR1 (155 bp; Table 3). Blots were scanned using the program ImageQuant 5.2 Molecular Dynamics.

### Gel Electrophoresis and Immunoblotting

Protein extracts obtained from R1158-1 *ALT1 ALT2-yECitrine* and R1158-2 *ALT1 tetO7ALT2-yECitrine ALT1*-TAP, *ALT2*-TAP were subjected to SDS-PAGE on 10% slab gels and transferred to nitrocellulose membranes. Incubation with antibodies was carried out as described previously [51]. For the TAP-tagged strains, anti-TAP rabbit polyclonal antibody (CAB 1001; OPEN Biosystems) was used at a 1∶5,000 dilution. Anti-Rabbit HRP antibody (Santa Cruz Biotechnology Inc.) was diluted 1∶10,000. For the yECitrine-tagged strains, anti-GFP mouse Roche monoclonal antibody (Cat. No. 11 814 460 001) was used at a 1∶5,000 dilution. Peroxidase-conjugated anti-mouse antibodies were diluted 1∶10,000. Immunoblot signaling was optimized by analyzing a number of combinations of antigen and antibody concentrations in the linear range of detectability. As a loading control all membranes were immunoblotted using a 1∶7,500 dilution of mouse anti-Lys20/Lys21 antibody. Anti mouse HRP-conjugated antibody was diluted 1∶12,500.

### Chromatin Immunoprecipitation (ChIP)

Formaldehyde cross-linking and immunoprecipitations were carried out by the procedure described by Hecht *et al*. (1995) [52]. Yeast cells (150 ml of OD_600_ 0.3 and 2.0 from glucose-alanine grown cultures) were incubated on ice for 15 min and cross-linked with 1% formaldehyde for 60 min at room temperature. After addition of 125 mM glycine and incubation for 5 min with gentle agitation, cells were harvested and washed with saline Tris-buffer. Pelleted cells were suspended in lysis buffer (140 mM NaCl, 1 mM EDTA, 50 mM HEPES/KPH, 1% Triton X-100, 0.1% sodium deoxycholate) with protease inhibitor cocktail (Complete Mini, Roche). Cells were lysed with glass beads, and collected by centrifugation. Extracts were sonicated to produce chromatin fragments of 1000 bp, with average size of 500 bp. ChIP was conducted with 1 µg anti-c-Myc antibody (9E 11, Santa Cruz Biotechnology) for 3 h, washed, suspended in 1 X TE/1% SDS and incubated overnight at 65°C in order to reverse the formaldehyde cross-linking. The immunoprecipitates were incubated with proteinase K (Roche) followed by phenol/chloroform/isoamyl alcohol extraction, precipitated and suspended in 1 X TE buffer. The primer sets used for PCR analysis are listed in Table 3. PCR products were resolved on a 1.5% agarose gel stained with ethidium bromide.

### Cell Extract Preparation and Alanine Aminotransferase Enzymatic Assay

Yeast cells were grown to O.D._600 nm_ 1.0, harvested by centrifugation, and washed with cold water. Pellets were suspended in cold extraction buffer (50 mM HEPES, 1 mM PMSF, 1 mM EDTA, 1 mM DTT ) and mechanically disrupted with glass beads. The resulting extract was centrifuged to eliminate cellular debris (5,000 rpm, 4°C, 15 min). The supernatant was recovered, and pyridoxal-5-phosphate was added to a 100 µM final concentration. Enzymatic assay was a modified version from García-Campusano [20]. The reaction mixure contained reaction buffer (pH8 50 mM Tris-HCl, 4 mM MgCl_2_, 150 mM KCl) 400 mM alanine, 24 mM α-ketoglutarate, 250 µM NADH, 40 µM pyridoxal 5-phosphate and 5 U/mL of lactate dehydrogenase. For Alt2 activity assays were performed at various combinations of pH (5–9), alanine (100–500 mM) and α-ketoglutarate (1.5- 24 mM) concentrations, extracts were also dialyzed and no activity was detected. As control assays were performed without alanine. To determine specific activity the slope obtained from this negative control was substracted to the complete assay. All assays were performed at 340 nm, 30°C in a Varian Cary 50 spectrophotometer.

### Metabolite Extraction and Analysis

Cell-free extracts and samples for intracellular amino acid determination were prepared as previously described [36].

### Phylogenetic Analyses of Alanine Aminotransferases

The evolutionary history of *ALT1* and *ALT2* in post-WGD yeasts was inferred using the Maximum-Likelihood method based on the JTT matrix-based model [29]. The tree with the highest log likelihood (−6922.5111) is shown. Initial tree(s) for the heuristic search were obtained automatically by applying Neighbor-Join and BioNJ algorithms to a matrix of pairwise distances estimated using a JTT model, and then selecting the topology with superior log likelihood value. The tree is drawn to scale, with branch lengths measured in the number of substitutions per site. The analysis involved 14 amino acid sequences.The analysis involved 15 aminoacid sequences. All positions containing gaps and missing data were eliminated. Evolutionary analyses were conducted in MEGA5 [30], [31].

### Amino-acid and Promoter Multiple Alignment

Promoter sequences were obtained from http://www.broad.mit.edu/annotation/fungi/comp_yeasts/downloads.html. Amino-acid sequences were obtained from http://www.broadinstitute.org/regev/orthogroups/. Multiple sequence alignment was performed using Clustal W.

## Supporting Information

Figure S1 ***ALT1*** **and** ***ALT2*** **display differential gene expression pattern.** Northern blot of total RNA prepared from *ALT1*-TAP and *ALT2*-TAP strains grown on either glucose-ammonium (A) or glucose-alanine (B). Samples were collected from various OD_600_ as stated. Representative results from three experiments are shown.
(TIF)

Figure S2 **The** ***ALT1*** **and** ***ALT2*** **promoters have consensus Nrg1-binding sites.** Full promoters are depicted as rectangles with *cis*-acting presumed binding sites for Nrg1, depicted as bars. Conserved binding sites are shaded in black while non-conserved site is depicted as white symbol, according to the multiple alignment of three yeast species performed for each promoter region (Figs S3 and S4). Binding sites are numbered starting from the most 5′and the number is placed at the 3′end of each site.
(TIF)

Figure S3 ***ALT1*** **promoter complete sequence.** Multiple alignment of three yeast species shows a single conserved Nrg1 presumed binding site.
(TIF)

Figure S4 ***ALT2*** **promoter complete sequence.** Multiple alignment of three yeast species shows a single consensus Nrg1 presumed binding site which is not fully conserved when promoter sequence of three yeast species were aligned.
(TIF)

Figure S5 ***ALT2*** **Multiple alignment of Alt proteins from post-WGD.** Multiple alignment of Alt proteins from post-WGD yeasts shows that the 11 crucial catalytic residues involved in cofactor and substrate binding (shaded) have been conserved in all cases [32]. Alt2 loss of function could be attributed to mutations affecting folding or oligomerization.
(TIF)
