# Supplementary figures and images for: Paralogous ALT1 and ALT2 Retention and Diversification Have Generated Catalytically Active and Inactive Aminotransferases in Saccharomyces cerevisiae
Source: PLoS One. 2012 Sep 25;7(9):e45702. doi: 10.1371/journal.pone.0045702 (PMC3458083; doi:10.1371/journal.pone.0045702)

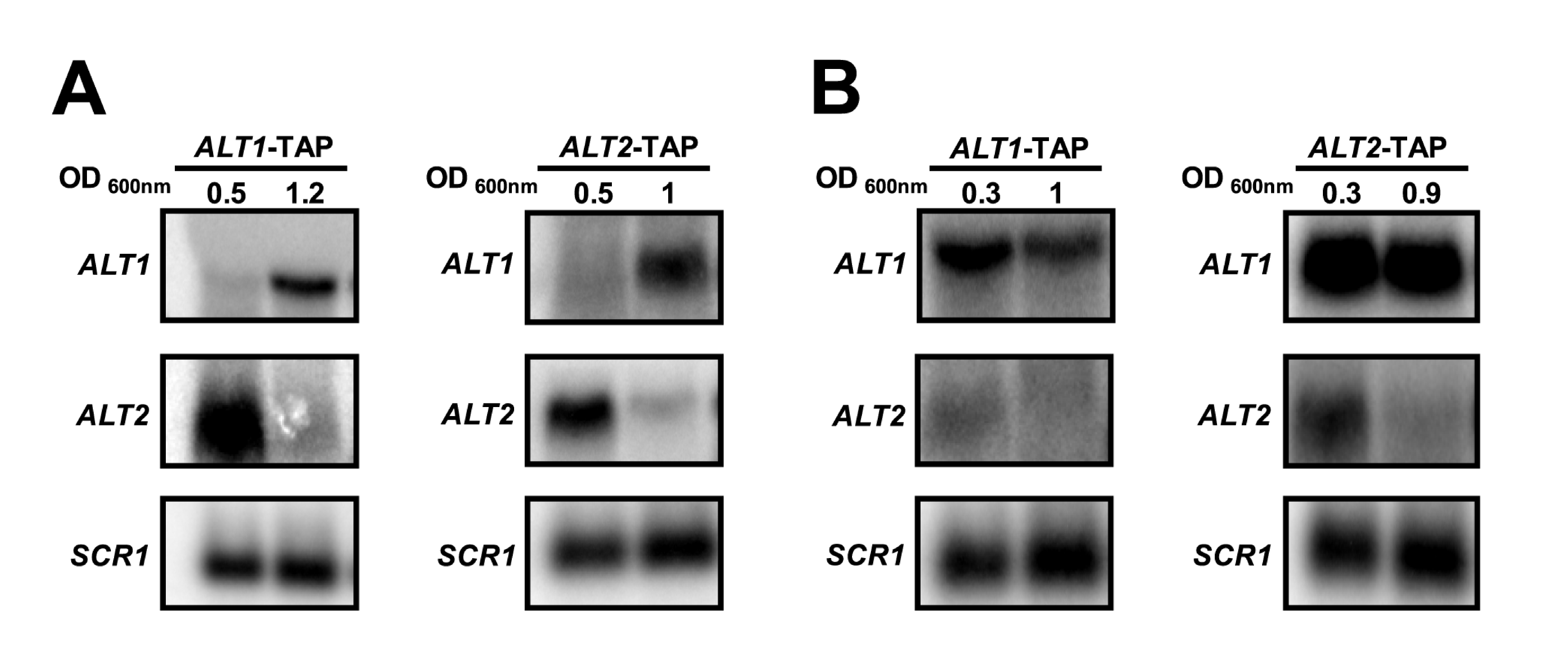

Supplement: Figure S1 — ALT1 and ALT2 display differential gene expression pattern. Northern blot of total RNA prepared from ALT1-TAP and ALT2-TAP strains grown on either glucose-ammonium (A) or glucose-alanine (B). Samples were collected from various OD600 as stated. Representative results from three experiments are shown. (TIF) [file pone.0045702.s001.tif]

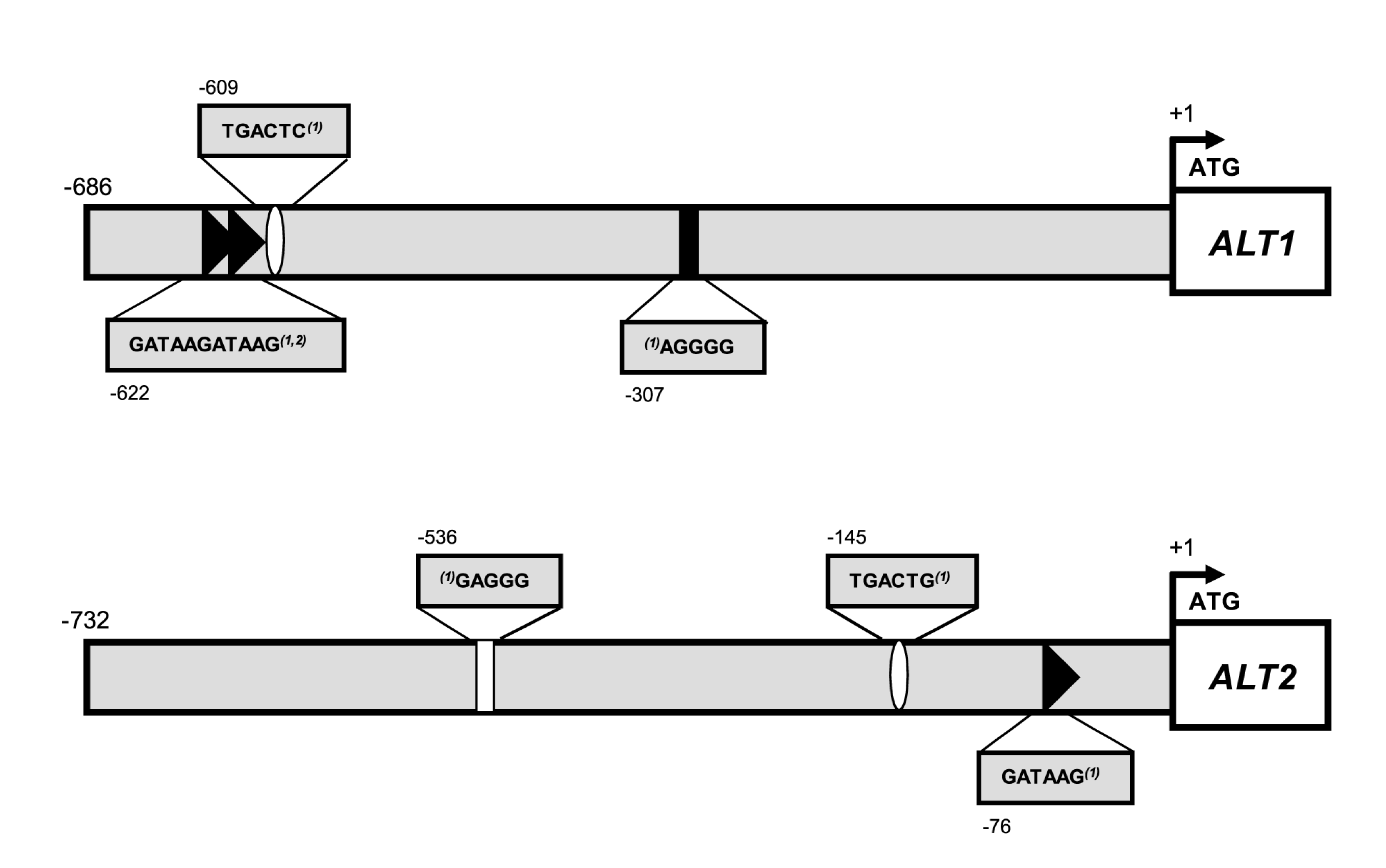

Supplement: Figure S2 — The ALT1 and ALT2 promoters have consensus Nrg1-binding sites. Full promoters are depicted as rectangles with cis-acting presumed binding sites for Nrg1, depicted as bars. Conserved binding sites are shaded in black while non-conserved site is depicted as white symbol, according to the multiple alignment of three yeast species performed for each promoter region (Figs S3 and S4). Binding sites are numbered starting from the most 5′and the number is placed at the 3′end of each site. (TIF) [file pone.0045702.s002.tif]

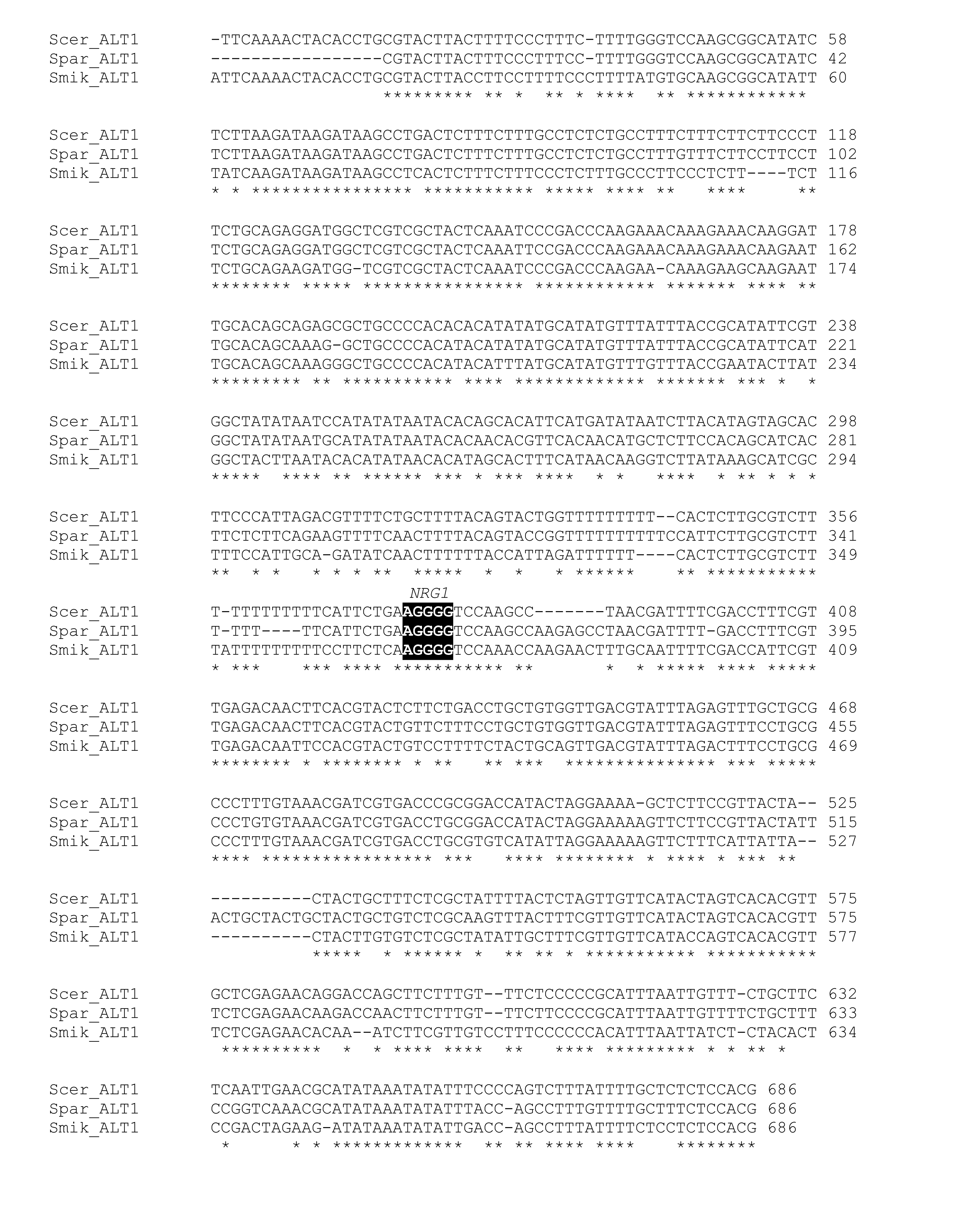

Supplement: Figure S3 — ALT1 promoter complete sequence. Multiple alignment of three yeast species shows a single conserved Nrg1 presumed binding site. (TIF) [file pone.0045702.s003.tif]

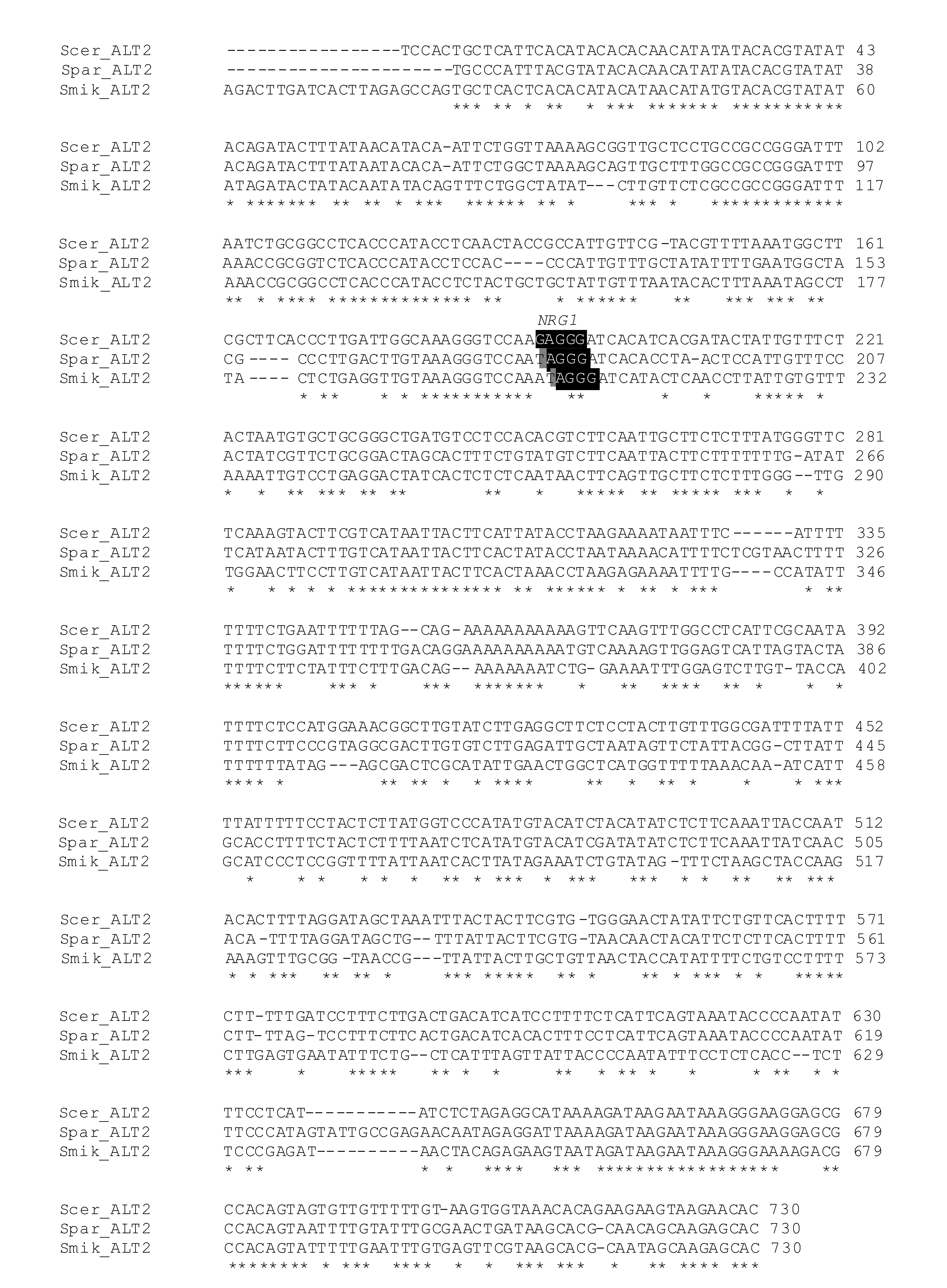

Supplement: Figure S4 — ALT2 promoter complete sequence. Multiple alignment of three yeast species shows a single consensus Nrg1 presumed binding site which is not fully conserved when promoter sequence of three yeast species were aligned. (TIF) [file pone.0045702.s004.tif]

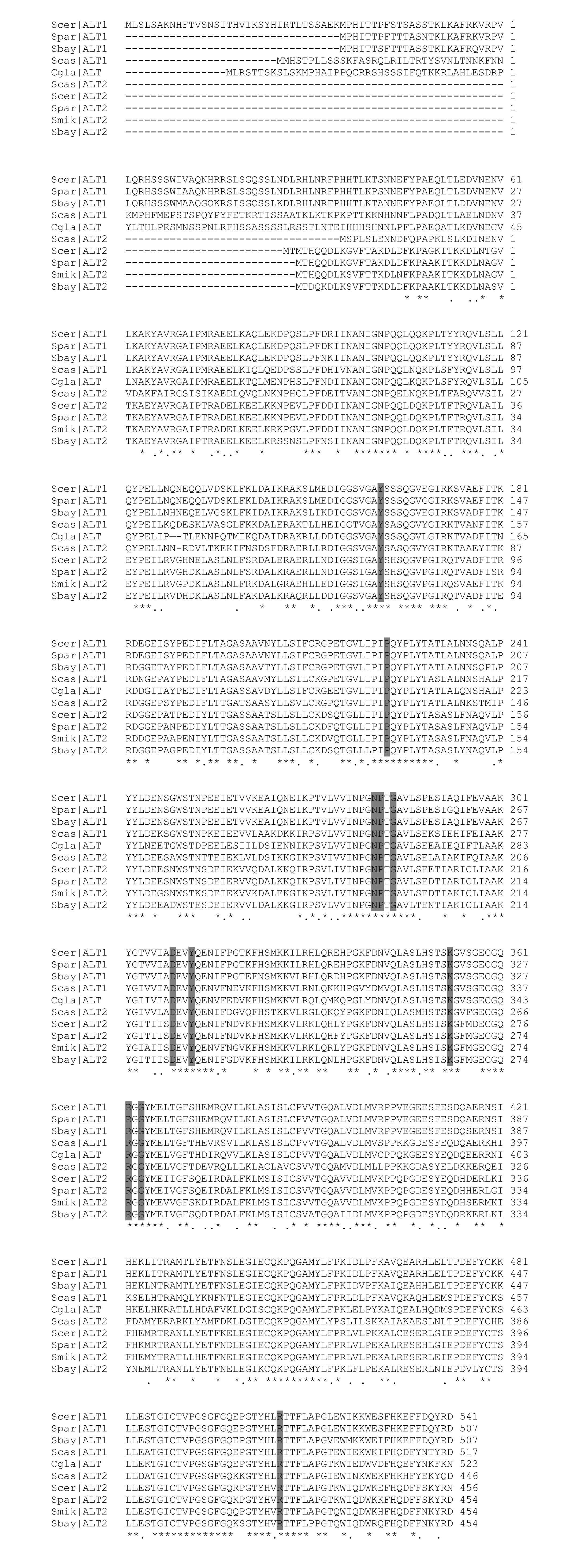

Supplement: Figure S5 — ALT2 Multiple alignment of Alt proteins from post-WGD. Multiple alignment of Alt proteins from post-WGD yeasts shows that the 11 crucial catalytic residues involved in cofactor and substrate binding (shaded) have been conserved in all cases [32]. Alt2 loss of function could be attributed to mutations affecting folding or oligomerization. (TIF) [file pone.0045702.s005.tif]
